# A method for assessing robustness of the results of a star-shaped network meta-analysis under the unidentifiable consistency assumption

**DOI:** 10.1186/s12874-021-01290-1

**Published:** 2021-06-01

**Authors:** Jeong-Hwa Yoon, Sofia Dias, Seokyung Hahn

**Affiliations:** 1 Interdisciplinary Program in Medical Informatics, Seoul National University College of Medicine, Seoul, South Korea; 2 Institute of Health Policy and Management, Medical Research Center, Seoul National University, Seoul, South Korea; 3 Centre for Reviews and Dissemination, University of York, York, UK; 4 Department of Human Systems Medicine, Medical Statistics Laboratory, Seoul National University College of Medicine, 103 Daehak-ro, Jongno-gu, Seoul, 03080 South Korea

**Keywords:** Star-shaped network, Indirect comparisons, Network meta-analysis, Inconsistency, Sensitivity analysis, Data imputation

## Abstract

**Background:**

In a star-shaped network, pairwise comparisons link treatments with a reference treatment (often placebo or standard care), but not with each other. Thus, comparisons between non-reference treatments rely on indirect evidence, and are based on the unidentifiable consistency assumption, limiting the reliability of the results. We suggest a method of performing a sensitivity analysis through data imputation to assess the robustness of results with an unknown degree of inconsistency.

**Methods:**

The method involves imputation of data for randomized controlled trials comparing non-reference treatments, to produce a complete network. The imputed data simulate a situation that would allow mixed treatment comparison, with a statistically acceptable extent of inconsistency. By comparing the agreement between the results obtained from the original star-shaped network meta-analysis and the results after incorporating the imputed data, the robustness of the results of the original star-shaped network meta-analysis can be quantified and assessed. To illustrate this method, we applied it to two real datasets and some simulated datasets.

**Results:**

Applying the method to the star-shaped network formed by discarding all comparisons between non-reference treatments from a real complete network, 33% of the results from the analysis incorporating imputed data under acceptable inconsistency indicated that the treatment ranking would be different from the ranking obtained from the star-shaped network. Through a simulation study, we demonstrated the sensitivity of the results after data imputation for a star-shaped network with different levels of within- and between-study variability. An extended usability of the method was also demonstrated by another example where some head-to-head comparisons were incorporated.

**Conclusions:**

Our method will serve as a practical technique to assess the reliability of results from a star-shaped network meta-analysis under the unverifiable consistency assumption.

**Supplementary Information:**

The online version contains supplementary material available at 10.1186/s12874-021-01290-1.

## Background

Network meta-analyses based on systematic reviews are often used to produce evidence for medical decision-making, such as deciding which of various treatment options is the best for a pre-defined population of patients. Specifically, network meta-analysis is a statistical method for integrating the data available from a network of multiple randomized controlled trials (RCTs) that involve multiple interventions, to estimate their relative effects by comparing them directly, indirectly, or both [1, 2]. The objective of a network meta-analysis is to compare the relative efficacy and/or safety of multiple medical interventions and to rank each treatment for a corresponding outcome [3].

Since a network meta-analysis combining all information from RCTs on multiple interventions provides an internally coherent set of estimates of the relative treatment effects between competing interventions [4–6], the included trials should be comparable; that is, there should be no imbalance in the distribution of potential effect modifiers across the trials [7–9]. In principle this should ensure consistency of evidence, however the assumption of consistency across direct and indirect evidence should also be statistically checked [10–12]. When the assumption of consistency is satisfied, a network meta-analysis may have acceptable validity, whereas this will be questionable when inconsistency, characterized by a discrepancy between direct and indirect evidence, is found [13]. However checking the consistency of the direct and indirect evidence in a network is only feasible when there are one or more closed loops within an evidence network. A closed loop refers to a part of a network where each comparison has both direct and indirect evidence [14]. Methods of testing for inconsistency in a network have been previously presented, and are distinguished by how to treat inconsistency [10–12, 15–17]. If the consistency assumption is violated in a network, a further qualitative evaluation is necessary to identify its sources [7–9].

However, researchers might encounter an evidence network where all treatments have been compared only with a common treatment, but not with each other. For example, new drugs are often compared with placebo or standard care, rather than to active treatments, in trials conducted for the purpose of obtaining approval for drug licensing [18]. Once a drug receives regulatory approval, there may no longer be any commercial incentive to compare the drug against other alternatives, and therefore there are occasions where no head-to-head trials between active treatments exist [19]. Such networks do not have any closed loops, and are referred to as ‘star-shaped networks’ [20]. A study reported that 47 (31%) of 152 network analyses published in PubMed between inception and March 2011 included star-shaped networks [21]. Although a decade has passed since then, many network meta-analyses still consist of interventions that do not have both indirect and direct comparisons or are conducted in contexts where one or few closed loops are available. For example, with advances of biologics for the treatment of rheumatoid arthritis over the past two decades, its evidence network, which included only indirect evidence in the first decade, has now incorporated some (albeit few) head-to-head comparisons [22]. In a star-shaped network, statistically detecting or checking inconsistency is impossible, thus researchers need to rely solely on a qualitative evaluation that studies are comparable, before integrating the data into a network meta-analysis under the consistency assumption [23–25]. However, there may be a certain degree of inconsistency between the evidence from the included indirect comparisons and the unknown direct comparisons; it may be impossible to detect statistically, but should nonetheless be considered. Therefore, it is necessary to explore the degree to which results from a star-shaped network are robust to potential inconsistencies.

In this article, we suggest a sensitivity analysis for evaluating the robustness of the results of a star-shaped network meta-analysis, and illustrate some examples of applying the method to two real datasets and four simulated datasets. We then provide an interpretation of the results for each example. We finally discuss the proposed method and its usability.

## Method development

### Notation, models, and method of testing for inconsistency

Let ${\hat{\theta}}_{ijk}$ be the observed relative effect size of treatment *k* (*k= T*_2_, ⋯, *T*_*p*_*)* compared to treatment *j* (*j= T*_0_, ⋯, *T*_*p* − 1_) from the *ith* study comparing treatment *j* versus *k* where a network contains *p* + 1 treatments *T*_0_, ⋯, *T*_*p*_, with ${\hat{\theta}}_{ijk}$ following a normal distribution, $N\Big({\theta}_{ijk},{\sigma}_{ijk}^2$). The parameter *θ*_*ijk*_ is the study-specific treatment effect of treatment *k* relative to *j* in study *i*. It is conventional that the estimated variance of ${\hat{\theta}}_{ijk}$, $\hat{\mathit{\operatorname{var}}}\left({\hat{\theta}}_{ijk}\right)$, is treated as if it were the true variance ${\sigma}_{ijk}^2$ [26, 27]. The distribution is thus assumed to satisfy ${\hat{\theta}}_{ijk}\sim N\left({\theta}_{ijk},\hat{\mathit{\operatorname{var}}}\left({\hat{\theta}}_{ijk}\right)\right)$. A model of *θ*_*ijk*_ is as follows:

$$ {\theta}_{ijk}\sim Normal\left({d}_{jk},{\tau}^2\right). $$

Here, *d*_*jk*_ is the mean study-specific effect size of treatment *k* compared to treatment *j*. We used a usual random-effects model [28, 29], which allows for between-study variation (*τ*^2^) that is common for all comparisons in a network. For simplicity, the between-study variation is assumed to be identical across all contrasts; however, between-study variation can also be modeled separately for each contrast [11, 30].

In the standard approach of performing a network meta-analysis, the basic parameters ${d}_{T_0k}$ and ${d}_{T_0j}$ (*j* and *k* ≠ *T*_0_) are first defined using a chosen reference intervention (*T*_0_), which is usually placebo or a conventional treatment [31]. The functional parameter *d*_*jk*_ (*j* and *k* ≠ *T*_0_) is then defined by a consistency relationship, ${d}_{jk}={d}_{T_0k}-{d}_{T_0j}$. A model based on this approach is called a ‘consistency model’. For a simple network with three interventions A, B, and C, the consistency model would estimate the basic parameters, *d*_*AB*_ and *d*_*AC*_, from all available evidence. The functional parameter, *d*_*BC*_, is calculated using the consistency equation, as *d*_*AC*_ − *d*_*AB*_. A full description of the model is given in Appendix 1 (Additional file 1) for this simple case. In addition, the network meta-analysis can rank all the treatments from best to worst [32].

**Fig. 1:**
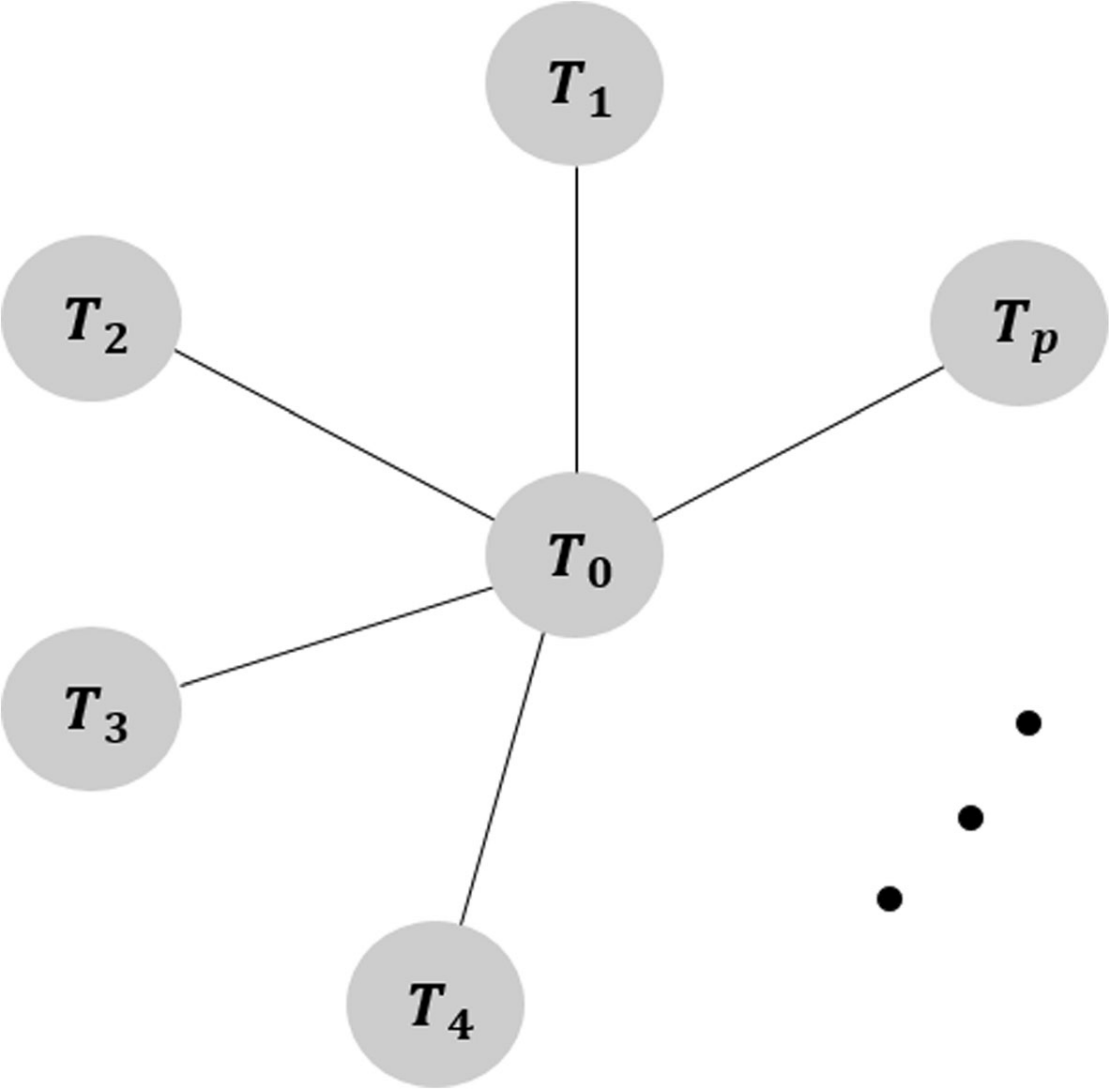
A graphical representation of a star-shaped network consisting of one common comparator treatment (*T*_0_) and *p* other alternative treatments (*T*_1_, ⋯, *T*_*p*_). Each node represents an intervention, and a link between two nodes reflects one or more randomized controlled trials

For a star-shaped network where only a common comparator (*T*_0_) is compared with all other alternative treatments (*T*_1_, ⋯, *T*_*p*_) without any head-to-head comparison among *T*_1_, ⋯, *T*_*p*_ as shown in Fig. 1, *T*_0_ is naturally assigned as the reference treatment in the above model for performing a network meta-analysis to estimate the basic parameters, ${d}_{T_0{T}_1}$, ⋯, ${d}_{T_0{T}_p}$. The relative effect sizes among the non-reference treatments are calculated by indirect comparisons.

An inconsistency model, in which consistency is not assumed, can be used to check whether the assumption of consistency holds [13]. This model represents each contrast between treatments in the network as an unrelated basic parameter estimated only from direct evidence; therefore, this is equivalent to conducting a separate pairwise meta-analysis with a shared heterogeneity parameter. For a fully connected simple network, when direct evidence on all contrasts is available, the inconsistency model would define the basic parameters, *d*_*AB*_, *d*_*AC*_, and *d*_*BC*_, without assuming any relationship between the parameters (see Additional file 1: Appendix 1). In contrast, if direct evidence is not available for one contrast, say BC, the model would estimate the basic parameters, *d*_*AB*_ and *d*_*AC*_, but the relative effect size between B versus C cannot be estimated. In a star-shaped network, there is no difference in fit or estimated treatment effects between consistency and inconsistency models because the basic parameters are defined identically in both models.

Consistency and inconsistency models can be fitted in a Bayesian framework using non-informative prior distributions for each defined parameter. Comparison of residual deviance and heterogeneity estimates between the two models can suggest inconsistency [13, 33]. When the inconsistency model produces the smallest residual deviance value, there is potential overall inconsistency in the corresponding evidence network. Similarly when the estimated heterogeneity is smaller in the inconsistency model than in the consistency model, this can suggest inconsistency. No particular cut-off value was considered for determining a meaningful difference.

### Statistical methods

We considered non-directly connected pairs in a star-shaped network as missing to conduct a sensitivity analysis. For a star-shaped network consisting of one common comparator as a reference treatment, and *p* non-reference treatments (Fig. 1), we filled in the hypothetical RCT data for all the *p*(*p* − 1)/2 missing pairwise comparisons, producing fully connected network (hereafter called a ‘complete network’). The imputed data consisted of study-level treatment effect sizes (${\hat{\theta}}_{ijk}^{\ast }$) and their variances ($\hat{\mathit{\operatorname{var}}}\left({\hat{\theta}}_{ijk}^{\ast}\right)$). They were generated to simulate a situation that would allow a mixed treatment comparison with some extent of inconsistency that is still acceptable statistically, where the acceptance was determined by examining whether a consistency model has a lower residual deviance value than an inconsistency model, so that the complete network resulting from imputation can be aggregated under the consistency assumption. By comparing the agreement between the analysis results from the original star-shaped network and the complete network, the robustness of the results of the original star-shaped network meta-analysis was assessed.

#### Imputation strategy

For the *p*(*p* − 1)/2 contrasts among non-reference treatments in the star-shaped network, the imputed data were generated to meet the following conditions:

- I: For each contrast between specific treatments, if the effect size estimated from the original star-shaped network is positive (or negative), the pooled effect size from a pairwise meta-analysis of the imputed data is assumed to be less (or greater) than that indirectly produced from the original star-shaped network meta-analysis. This condition is put in place to run the sensitivity analysis from a conservative point of view, assuming that the artificial direct estimate is smaller (or larger) than the observed indirect estimate.
- II: For each contrast, the precision of the pooled effect size from the pairwise meta-analysis of the imputed data is the same as the precision of the effect size indirectly estimated in the original star-shaped network meta-analysis. This means that the variance of individually imputed effect sizes will produce the maximal variance in their pooled effect size, since it is generally considered that indirectly estimated effect sizes have greater variance than direct estimates [30].
- III: For each contrast, the extent of heterogeneity in the imputed data for the effect size of the contrast is the same as that of the overall heterogeneity across contrasts in the star-shaped network. This assumption serves to maintain the level of overall heterogeneity in the network after imputation, enabling us to investigate only the impact of the potential extent of inconsistency on the results of the sensitivity analysis.

#### Assessing the robustness of conclusions from a star-shaped network meta-analysis through imputation

We illustrated the sensitivity analysis method using the simplest star-shaped network, which involved RCTs of A versus B and A versus C. The RCT data, ${\hat{\theta}}_{iAB}$ with $\hat{\mathit{\operatorname{var}}}\left({\hat{\theta}}_{iAB}\right)$ for *i* = 1, …, *N* and ${\hat{\theta}}_{iAC}$ with $\hat{\mathit{\operatorname{var}}}\left({\hat{\theta}}_{iAC}\right)$ for *i* = 1, …, *M*, are given, when *N* and *M* are the numbers of RCTs for A versus B and A versus C, respectively. From the star-shaped network meta-analysis, we obtained estimates of the basic parameters, ${\hat{d}}_{AB}$ and ${\hat{d}}_{AC}$, and an estimate of between-study variation, ${\hat{\tau}}^2$. The indirectly estimated effect size between B and C and its variance are ${\hat{d}}_{AC}-{\hat{d}}_{AB}$ and $\hat{\mathit{\operatorname{var}}}\left({\hat{d}}_{AC}-{\hat{d}}_{AB}\right)$, respectively.

We generated ${\hat{\theta}}_{iBC}^{\ast }$ and $\hat{\mathit{\operatorname{var}}}\left({\hat{\theta}}_{iBC}^{\ast}\right)$, with *i* = 1, …, *l* for *l* hypothetical RCTs comparing B and C using the imputation strategy described in the above section. The value of *l* was determined while calculating $\hat{\mathit{\operatorname{var}}}\left({\hat{\theta}}_{iBC}^{\ast}\right)$. The effect sizes ${\hat{\theta}}_{1 BC}^{\ast },\cdots, {\hat{\theta}}_{lBC}^{\ast }$ were generated from the following distributions:

$$ {\hat{\theta}}_{iBC}^{\ast}\sim N\left({\theta}_{iBC}^{\ast },\hat{\mathit{\operatorname{var}}}\left({\hat{\theta}}_{iBC}^{\ast}\right)\right),\mathrm{for}\ i=1,\dots, l. $$

The imputation parameters, ${\theta}_{1 BC}^{\ast }$, $\cdots, {\theta}_{lBC}^{\ast }$, were generated from a normal distribution, $N\left({\hat{d}}_{AC}-{\hat{d}}_{AB}+{\overline{\omega}}_{BC},{\hat{\tau}}^2\right)$. The constant ${\overline{\omega}}_{BC}$ was defined artificially to represent the extent of potential inconsistency between the direct (${\hat{\theta}}_{BC}^{\ast }$) and indirect (${\hat{d}}_{AC}-{\hat{d}}_{AB}$) evidence. Under condition I, if ${\hat{d}}_{AC}-{\hat{d}}_{AB}<0$, ${\overline{\omega}}_{BC}$ should be positive, and if ${\hat{d}}_{AC}-{\hat{d}}_{AB}>0$, ${\overline{\omega}}_{BC}$ should be negative.

The variances $\hat{\mathit{\operatorname{var}}}\left({\hat{\theta}}_{1 BC}^{\ast}\right)=\hat{\mathit{\operatorname{var}}}\left({\hat{\theta}}_{2 BC}^{\ast}\right)=\cdots =\hat{\mathit{\operatorname{var}}}\left({\hat{\theta}}_{lBC}^{\ast}\right)=l\bullet \hat{\mathit{\operatorname{var}}}\left({\hat{d}}_{AC}-{\hat{d}}_{AB}\right)-{\hat{\tau}}^2$ were calculated to satisfy the given conditions (II, III), and they were set up to be identical for simplicity (the derivation of this formula can be found in Additional file 1: Appendix 2). However, *l* was an arbitrarily chosen number, with the restriction that $l\bullet \hat{\mathit{\operatorname{var}}}\left({\hat{d}}_{AC}-{\hat{d}}_{AB}\right)$ was larger than ${\hat{\tau}}^2.$

To account for potential uncertainty in the prediction of unknown data for the missing comparisons in a star-shaped network, we used a multiple imputation approach. From the defined distribution, the complete network data with imputations were generated *m* times and each of the *m* complete networks was analyzed using the consistency model. The resulting estimate of each parameter with its variance and the estimated probability of each treatment being the best were obtained by Rubin’s rules [34, 35], and each treatment was then ranked using the obtained probabilities. When pooling by Rubin’s rules [34, 35], the estimate of each parameter is summarized by taking the average over estimates from all imputed *m* complete networks, and its variance is produced by incorporating both within-imputation and between-imputation variability.

The above processes were repeated, changing the value of $\mid {\overline{\omega}}_{BC}\mid$ to increase from zero until the complete network started to have a larger residual deviance value when the consistency model was applied than when the inconsistency model was applied, which produced a range of values for $\left|{\overline{\omega}}_{BC}\right|$ that can be considered statistically acceptable for a synthesis by network meta-analysis under the consistency assumption. The value of *m* was determined as the point where the two residual deviance curves crossed only once and never again, that is where the threshold value was stabilized. The proportion of $\mid {\overline{\omega}}_{BC}\mid$ values that resulted in a consistent ranking of treatments to that from the original star-shaped network meta-analysis was presented as a percentage, as an indicator of the sensitivity of the results to the degree of potential inconsistency. A “consistent ranking” meant that the order of the originally observed ranking was unchanged.

The sensitivity analysis may be generalized to a star-shaped network with more than three interventions by employing ${\overline{\omega}}_{jk}$ for *j* = *T*_1_, ⋯, *T*_*p* − 1_ and *k* = *T*_2_, ⋯, *T*_*p*_ (*j* ≠ *k*). We demonstrated this case with *p* = 3, where ${\overline{\omega}}_{jk}\ \mathrm{for}\ j={T}_1,{T}_2$ and *k* = *T*_2_, *T*_3_ (*j* ≠ *k*) were simultaneously changed by an identical magnitude from zero in their respective directions.

The developed method was implemented in R software (version 3.3.3) [36].

### Application to datasets

#### Illustration of the method: smoking cessation dataset

To illustrate how the method can be applied, a dataset was drawn from a published and well-studied network meta-analysis [11, 16, 37] comparing four smoking cessation treatments: no intervention (*NI*), self-help (*SH*), individual counseling (*IC*) and group counseling (*GC*). The relative effect was measured by the logarithm of the odds ratio for successful smoking cessation at 6–12 months. There were 24 RCTs including two three-arm trials. In the original analyses, both the global model fit statistics and the inconsistency *p*-value suggested no presence of inconsistency (Additional file 2: Table S1). The reported overall measure of inconsistency, taken as the variance of inconsistency factor, was 0.61; this value was smaller than the value of between-study heterogeneity (0.78), suggesting an acceptable extent of inconsistency. The posterior distributions of the direct estimates overlapped with those of the estimates obtained using indirect evidence for all contrasts [16].

**Fig. 2:**
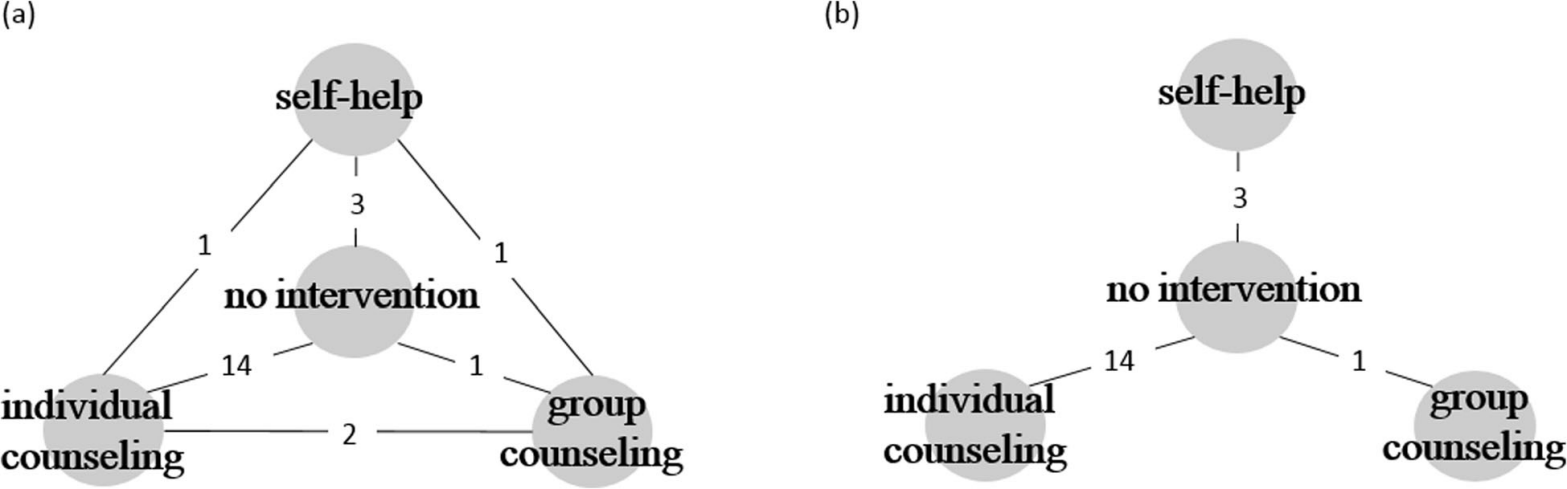
**a** A graphical representation of the evidence network for four smoking cessation counseling programs. **b** A graphical representation of the derived star-shaped network by eliminating four trials corresponding to direct comparisons among self-help, individual counseling, and group counseling. Each node represents an intervention, and a line between two nodes reflects one or more randomized controlled trials (RCTs). The numbers on each solid line connecting two interventions correspond to the number of RCTs comparing those interventions

In this exercise, we utilized only the 22 two-arm trials (Fig. 2a). A network meta-analysis was conducted using the consistency model to produce estimates of the basic parameters, *d*_*NI*, *SH*_, *d*_*NI*, *IC*_, and *d*_*NI*, *GC*_, where *NI* was the reference treatment. A ranking of the treatments was determined using the estimated probability for each treatment to be the best from this model.

We formed a star-shaped network by discarding data from the four RCTs that compared non-reference treatments head-to-head (Fig. 2b). For the intended star-shaped network, we initially performed a network meta-analysis using the consistency model. We subsequently applied the proposed method for sensitivity analysis.

From the sensitivity analysis, according to the absolute extent of inconsistency, $\left|{\overline{\omega}}_{jk}\right|$ (*j*= *SH*, *IC*, and *k* = *IC*, *GC*, *j* ≠ *k*), we plotted traces of residual deviances from the consistency and inconsistency models against the corresponding $\left|{\overline{\omega}}_{jk}\right|$ and indicated the point where those two curves crossed. Estimates of the basic parameters with their 95% credible intervals (CrIs), the probability that each treatment was the best for smoking cessation, and the treatment ranking were also plotted for each value of $\left|{\overline{\omega}}_{jk}\right|$ up to this point. The proportion of $\left|{\overline{\omega}}_{jk}\right|$ that resulted in a consistent ranking of treatments to that obtained from the star-shaped network meta-analysis was presented. To determine the number of imputations, we started with an imputation number of 100 and increased it by 100 until a stabilization of threshold was obtained at 500 imputations (Additional file 3: Figure S1).

#### Simulation for diverse scenarios

Datasets from a simple star-shaped network of RCTs of A versus B and A versus C were simulated according to levels of within- and between-study variability of treatment effect size (i.e., the standard errors of estimates from the individual trials and the extent of overall heterogeneity across contrasts) (see Additional file 2: Table S2). For each dataset, the number of trials for each contrast was set to be five. The effect sizes for each contrast were arbitrarily chosen to be a specified value when they were pooled, and to have a specific level of heterogeneity that was determined in terms of the *I*^2^ statistic. This statistic was used under the assumption that the effect sizes were normally distributed sample means. To consider differences in the treatment effect among the three interventions, the pooled treatment effect sizes for the comparisons (A versus B and A versus C) were set at 0.5 and 1 for the effect size of one alternative treatment relative to the reference treatment to be half of that of another alternative treatment relative to the reference treatment. We then generated individual trial-level effect sizes with their standard errors to comply with the condition that the probability for each treatment group being the best would be 0.66 for C, 0.33 for B, and 0 for A, respectively, while no heterogeneity existed. Starting from this basic scenario, we modified the level of standard error by halving it or by multiplying it by $\sqrt{2}$, which corresponds to the impact of doubling the variance while attempting to increase the scale of heterogeneity to the severe level. The considered values of the *I*^2^ statistic were 0% (no heterogeneity), 40% (moderate heterogeneity), and 70% (severe heterogeneity) [38].

This method was applied to each dataset. According to the absolute extent of inconsistency, represented by $\left|{\overline{\omega}}_{BC}\right|$, we plotted traces of residual deviances from the consistency and inconsistency models, and then indicated the point where those curves crossed. The probability of each treatment group being the most effective was plotted for each value of $\left|{\overline{\omega}}_{BC}\right|$ up to this point. The proportion of $\left|{\overline{\omega}}_{BC}\right|$ values that resulted in a ranking of treatments consistent with the original ranking in the star-shaped network was presented. For each simulated dataset, we ran the process by applying a sufficiently large number of imputations (500).

#### Extension of application: Crohn’s disease dataset

We demonstrated the extended usability of our method by considering network meta-analyses that are conducted in contexts where few closed loops are available. From an original network in a recently published review conducted to compare the effects of interventions for the maintenance of surgically induced remission in Crohn’s disease [39], a sub-network consisting of placebo, purine analogues, 5-aminosalicylic acid (5-ASA), adalimumab, and infliximab was abstracted (see Additional file 3: Figure S2 (a)). The relative effect was measured by the logarithm of the risk ratio for clinical relapse.

We plotted traces of residual deviances from the consistency and inconsistency models according to $\left|{\overline{\omega}}_{jk}\right|$ (*j* = placebo, *k* = adalimumab, infliximab, and *j* = 5 − ASA, *k* = infliximab), with an indication of the point where those two curves crossed. Since purine analogues were most frequently connected with other alternative treatments in the network, we chose them as the reference treatment. The estimates of the basic parameters and the probability to be the best treatment for reducing relapse were also plotted for each value of $\left|{\overline{\omega}}_{jk}\right|$ up to this point. The proportion of $\left|{\overline{\omega}}_{jk}\right|$ values that resulted in a consistent ranking of treatments compared to that obtained from the star-shaped network meta-analysis was presented. Since the example dataset contained two three arm trials, we used the shared parameter model [31] to incorporate both the arm-level and the trial-level data into the analysis. We set the number of imputations to 500.

## Results of application

### Smoking cessation dataset

When the consistency model was applied to the complete network, the resulting values for ${\hat{d}}_{NI, SH}^c$, ${\hat{d}}_{NI, IC}^c$ and ${\hat{d}}_{NI, GC}^c$ were 0.43 (95% CrI, − 0.38 to 1.25), 0.73 (0.26 to 1.20), and 1.38 (0.25 to 2.5), respectively, and the best treatment for smoking cessation was *GC*, followed by *IC*, *SH*, and *NI* (Additional file 2: Table S3). The star-shaped network formed by discarding the head-to-head contrast data produced ${\hat{d}}_{NI, SH}^s$, ${\hat{d}}_{NI, IC}^s$, and ${\hat{d}}_{NI, GC}^s$ values of 0.33 (− 0.73 to 1.39), 0.72 (0.19 to 1.25), and 3.52 (0.12 to 6.93), respectively, with the same order of ranking*.* However, the estimate, ${\hat{d}}_{NI, GC}^s$, which was obtained only from direct evidence, was more exaggerated than ${\hat{d}}_{NI, GC}^c$, and the probability of *GC* being the best intervention for smoking cessation became even higher.

**Fig. 3:**
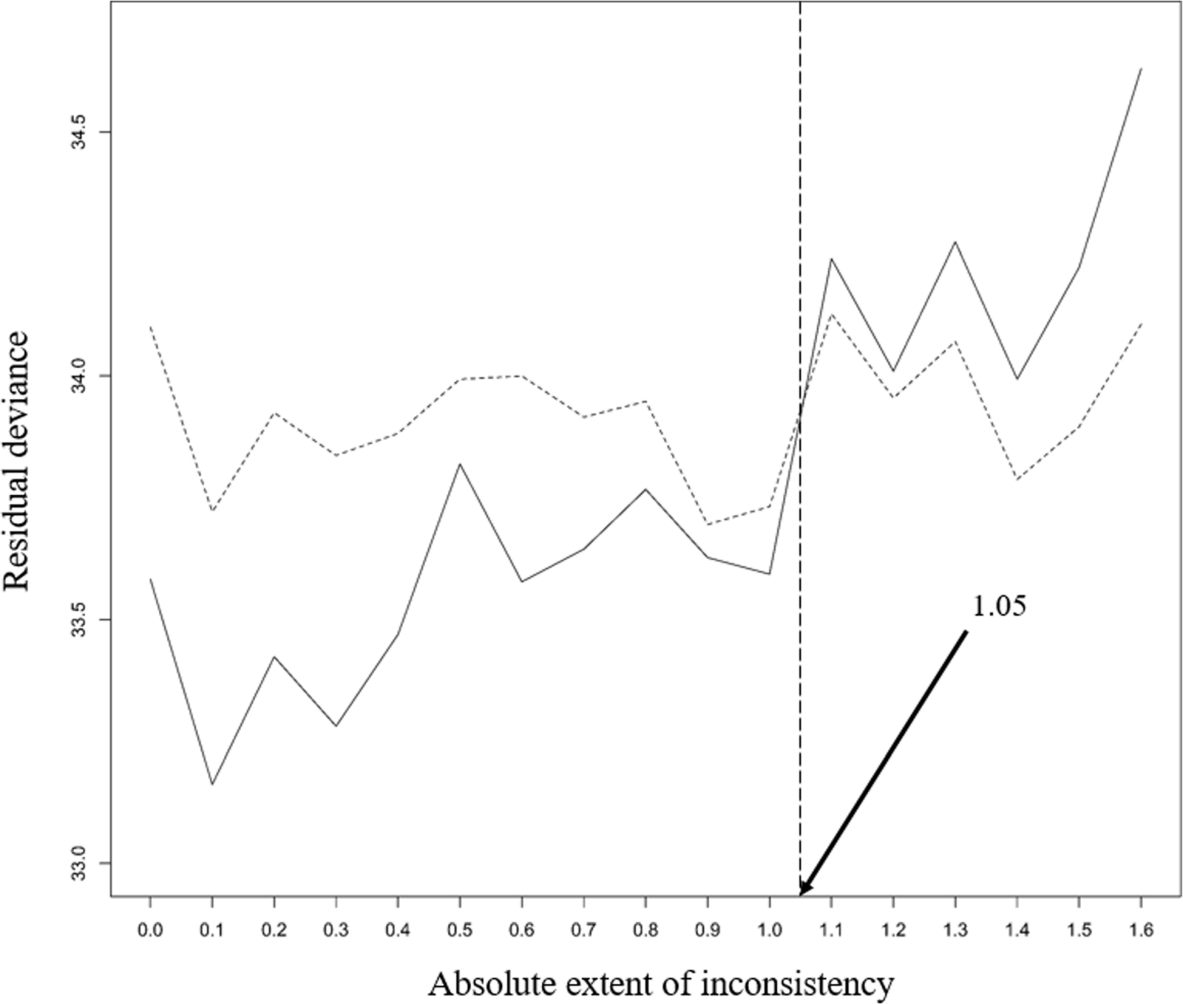
Residual deviances by model type (y-axis) against the absolute extent of inconsistency (x-axis). The solid line and dashed line indicate the consistency model and inconsistency model, respectively. A vertical line marks the point at which the two lines cross, and the value of that point on the x-axis is shown

**Fig. 4:**
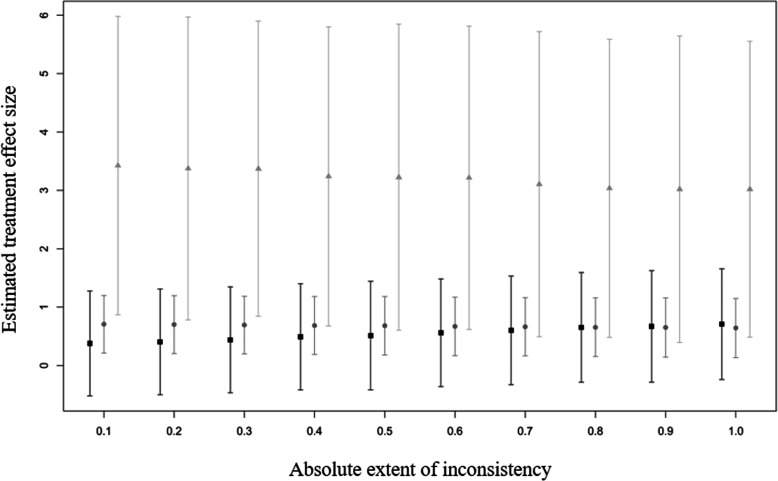
Interval plot of estimates of basic parameters against the extent of inconsistency (x-axis) within the obtained range. The black square, gray circle, and dim gray triangle symbols indicate the estimated treatment effect sizes for self-help, individual counseling, and group counseling compared to no intervention, with the vertical lines extending from the symbols representing 95% credible intervals

**Fig. 5:**
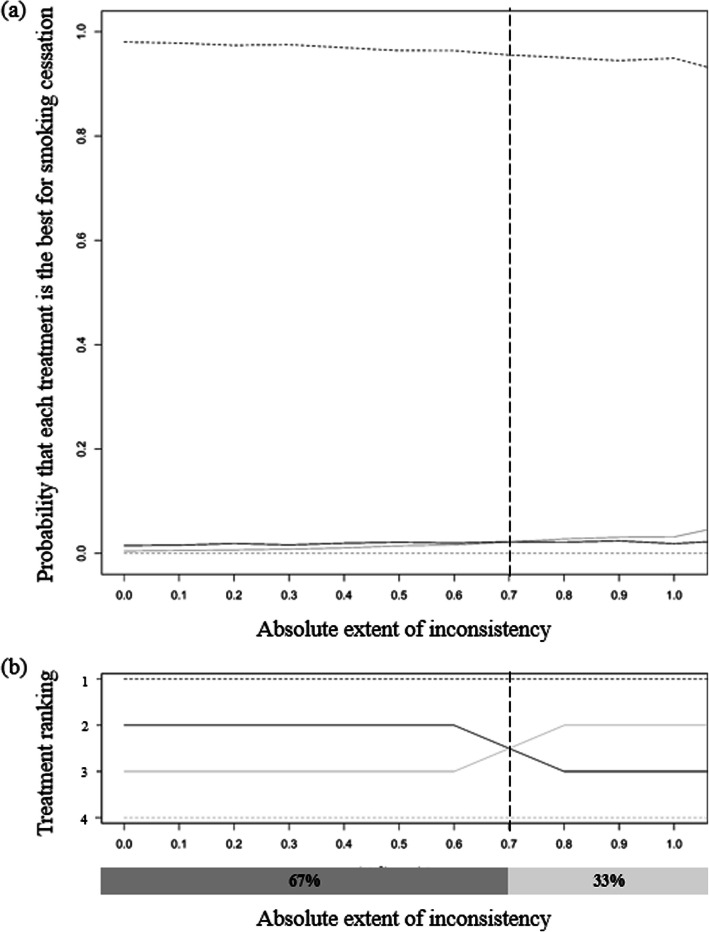
**a** Probability that each treatment is the best for smoking cessation against the extent of inconsistency within the obtained range. **b** Ranking of each treatment for successful smoking cessation against the extent of inconsistency within the obtained range. The gray dotted, gray solid, black solid, and black dotted lines indicate the probabilities and rankings corresponding to no intervention, self-help, individual counseling, and group counseling, respectively. A vertical line marks the point at which some lines cross, and the percentages in the dark gray and dim gray boxes represent the proportions of $\left|{\overline{\omega}}_{jk}\right|$ that resulted in a consistent ranking and an inconsistent ranking of treatments relative to the original ranking, respectively

The range of $\left|{\overline{\omega}}_{jk}\right|$ for statistically acceptable inconsistency was approximately from zero to 1.05 (Fig. 3), the upper threshold of which is a value in the middle of the half widths, 1.06, 0.53, 3.41, of the above intervals of ${\hat{d}}_{NI, SH}^s$, ${\hat{d}}_{NI, IC}^s$, and ${\hat{d}}_{NI, GC}^s$. As $\left|{\overline{\omega}}_{jk}\right|$ increased, the estimate of *d*_*NI*, *SH*_ increased and the estimate of *d*_*NI*, *GC*_ decreased (Fig. 4). The estimates of basic parameters became closer to each other, and the exaggerated probability of GC being the best intervention decreased to a level similar to the findings obtained from the original complete network (Fig. 5a), and the order of the ranking then changed (Fig. 5b). The proportion of $\left|{\overline{\omega}}_{jk}\right|$ values that produced a treatment ranking consistent with that from the star-shaped network meta-analysis was approximately 67%.

### Simulated datasets

**Fig. 6:**
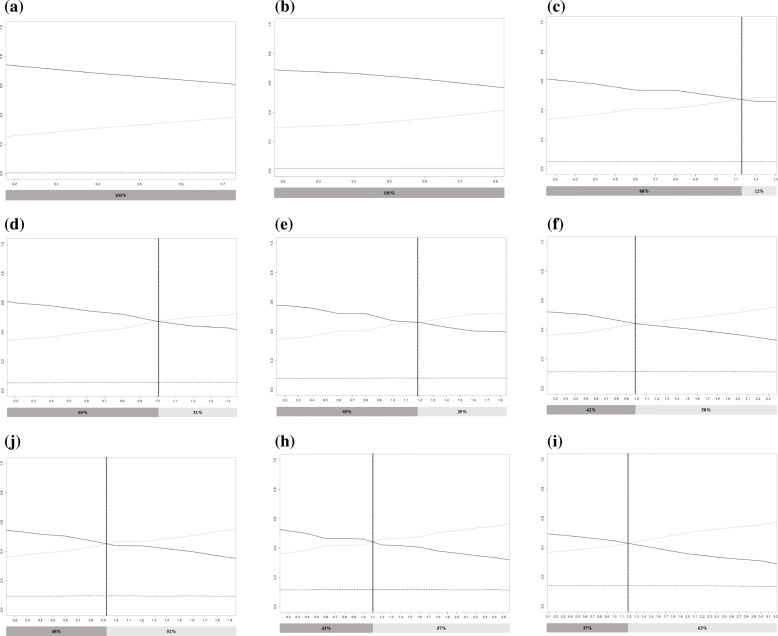
Probability of each group being the best (y-axis) against the extent of inconsistency, $\left|{\overline{\omega}}_{BC}\right|$ (x-axis), within the obtained range for each data set. **a** when *I*^2^ is 0% and the standard error is 1, **b** when *I*^2^ is 40% and the standard error is 1, **c** when *I*^2^ is 70% and the standard error is 1, **d** when *I*^2^ is 0% and the standard error is 2, and **e** when *I*^2^ is 40% and the standard error is 2, **f** when *I*^2^ is 70% and the standard error is 2, **g** when *I*^2^ is 0% and the standard error is $2\sqrt{2}$, **h** when *I*^2^ is 40% and the standard error is $2\sqrt{2}$, and **i** when *I*^2^ is 70% and the standard error is $2\sqrt{2}$. The black dotted, gray solid, and black solid lines indicate the probability corresponding to groups **a**, **b**, and **c**, respectively. A vertical line marks the point at which some lines cross, and the percentages in the dark gray and dim gray boxes represent the proportions of $\left|{\overline{\omega}}_{BC}\right|$ that resulted in a consistent ranking and an inconsistent ranking of treatments relative to the original ranking, respectively

For a given effect size, a star-shaped network with a greater level of between-study (or within-study) variability, when the level of within-study (or between-study) variability was fixed, produced a larger threshold of $\left|{\overline{\omega}}_{AB}\right|$ at which the residual deviance curves from the two models intersected (Additional file 3: Figure S3). The threshold showed that a greater extent of uncertainty present in an evidence network allowed a higher level of actual inconsistency to be acceptable. Within the range extending up to the threshold, the proportion of $\left|{\overline{\omega}}_{AB}\right|$ values that produced a consistent ranking of the treatments with the original ranking was smaller (Fig. 6). A small proportion indicates that the conclusions from the complete networks, simulated under assumption that there was no inconsistency, could have a great possibility of differing from the conclusions of the original star-shaped network.

In the network with the basic scenario, the proportion of $\left|{\overline{\omega}}_{BC}\right|$ values that produced a treatment ranking consistent with that from the star-shaped network meta-analysis was approximately 69% (Fig. 6d). In the absence of heterogeneity, when only the standard error was modified by halving it or to double the variance, the proportion increased to 100% and decreased to 48%, respectively (Fig. 6a and g). While keeping the level of standard error, as *I*^2^ increased to 40% and then to 70%, the proportion decreased to 65 and 42%, respectively (Fig. 6e and f).

### Crohn’s disease dataset

The range of $\left|{\overline{\omega}}_{jk}\right|$ for statistically acceptable inconsistency was zero to approximately 1.7 (see Additional file 3: Figure S2 (b)), where the obtained maximum value was located roughly in the middle of the half widths of the originally estimated 95% CrIs of the four basic parameters. As $\left|{\overline{\omega}}_{jk}\right|$ increased, the estimates of basic parameters became closer to each other, but none were reversed in ranking (Additional file 3: Figure S2 (c)). The proportion of $\left|{\overline{\omega}}_{jk}\right|$ values that produced a treatment ranking consistent with that from the original network meta-analysis was then 100% (Additional file 3: Figure S2 (d)). This can therefore strengthen confidence in the results from the original network meta-analysis.

## Discussion

In practice, we occasionally come across a situation where health technologies of interest have never been compared against each other, but it is still necessary to assess their comparative effectiveness based only on a star-shaped network meta-analysis under the unverifiable consistency assumption. We have developed a method for sensitivity analysis that accounts for an unknown degree of inconsistency by imputing data for all missing pairwise comparisons in a star-shaped network.

We established the imputation strategy based on the following rationale. If the effect size for each contrast estimated from the original star-shaped network is positive (or negative), the pooled effect size from a pairwise meta-analysis of the imputed data is less (or greater) than that. We set up this condition to run the sensitivity analysis from a conservative perspective, as the observed treatment difference (from indirect information only) should be considered biased if the true difference is closer to the null or if the direction of the effect may be different. In reality, the true difference might be one that even strengthens the existing conclusion, but we did not deal with such cases, since they would then not be a cause for concern and therefore beyond our scope. We also assumed that the precision of the pooled effect size obtained from the pairwise meta-analysis of the imputed data would be equal to the precision of the effect size obtained indirectly from the original star-shaped network meta-analysis. This equality implies that the variance of individually imputed effect sizes will produce the maximal variance of their pooled effect size. This could be considered as the most conservative case. If some information is available on the precision of the unknown direct estimate, regarding how relatively small it could be, it is possible to take that information into account in the equation of $\hat{\mathit{\operatorname{var}}}\left({\hat{\theta}}_{iBC}^{\ast}\right)$ to the variance of indirectly obtained estimate as a proportion.

We established the extent of heterogeneity in the imputed effect sizes necessary for each missing contrast to have the same level as the overall heterogeneity in the original star-shaped network. Unless the numbers of studies within contrasts are sufficiently large, it may be hard to estimate the overall heterogeneity, and any existing heterogeneity could be dramatically exaggerated. To take such cases into account, our simulation study considered a condition with severe heterogeneity. Furthermore, in practice, the number of included studies in a network meta-analysis is often insufficient to precisely estimate the heterogeneity variance. In that case, we may consider informative priors for heterogeneity variance to incorporate some external evidence into the network meta-analysis model [40, 41] in our method as an attempt to overcome this problem.

In this method, for a star-shaped network consisting of one common comparator and *p* alternative treatments, we imputed data for *p*(*p* − 1)/2 missing contrasts. If *p* is 2, 3, 4, or 5, the number of contrasts for data imputation would be 1, 3, 6, or 10, respectively. When *p* ≥ 4, the number of missing contrasts becomes larger than the number of connected contrasts, meaning that the proportion of unknown information is high. Therefore, for a star-shaped network where *p* ≥ 4, it may not be recommended to apply this method because data imputation may inordinately neutralize the evidence contained in the star-shaped network. When the proportion of missing contrasts is relatively small in a network involving more than 4 alternative treatments, but including few head-to-head comparisons, our suggested method can be used, and we presented the extended usability of this method through the example using a Crohn’s disease dataset.

Since the unit of imputed data in a network meta-analysis is a trial, the proportion of missing information is usually higher than that in ordinary applications. Therefore, a large number of imputations are required to stabilize the results of the sensitivity analysis through a multiple imputation strategy [34, 35]. In another example of meta-regression, the number of imputations was increased to 100 [42]. In our approach, stabilization was defined as occurring once the residual deviance curves of two models crossed and never overlapped again. The number of imputed complete networks, *m*, should be determined during the analysis depending on the data. For the smoking cessation example, the exploratory results by different numbers of imputations (m = 100, 200, 300, 400 and 500) in Additional file 3: Figure S1 suggest that 500 was sufficient. We also explored the number with several simulated datasets to confirm that repeating imputations 500 times is sufficient to achieve stabilization. Some exploratory residual deviance plots demonstrate that a much smaller number, such as 100, may be enough (Additional file 3: Figure S4). However, we recommend just applying a large number, such as 500, rather than running the exploration process for choosing the number of imputations per dataset, which would save much greater computational intensity.

The imputed data consisted of study-level treatment effect sizes (${\hat{\theta}}_{ijk}^{\ast }$) and their variances ($\hat{\mathit{\operatorname{var}}}\left({\hat{\theta}}_{ijk}^{\ast}\right)$). We established the assumption that the variances of the effect sizes for each contrast would be identical. According to the conditions described in the “Imputation strategy” section, the variances were calculated so that, for each contrast, the variance of the pooled effect size of the imputed data would be the same as that of the indirectly estimated effect size from the original star-shaped network meta-analysis. Since it is the precision of pooled estimate of the imputed effect sizes that contributes to estimation of basic parameters in the resulting network meta-analysis after imputation, any combination of values for the individual variances is acceptable as long as the overall precision is satisfying the condition. In the same context, for each contrast, we allowed the number of trials (*l*) to be arbitrarily chosen under the restriction that $l\bullet \hat{\mathit{\operatorname{var}}}\left({\hat{d}}_{Ak}-{\hat{d}}_{Aj}\right)$ (*j* ≠ *k* ≠ *A*) is larger than ${\hat{\tau}}^2.$ A trade-off exists between *l* and $1/\hat{\mathit{\operatorname{var}}}\left({\hat{\theta}}_{ijk}\right)$.

Methods of testing the consistency assumption are distinguished by how to treat inconsistency. The Bucher method [15], the back-calculation method, and the node-splitting method [16] are local test methods that evaluate the inconsistency of each contrast that constitutes a network. Global test methods assess the comprehensive inconsistency of the network based on modeling. The types of models used for testing include a random-inconsistency Bayesian model [11], a design-by-treatment interaction model [17], and an inconsistency model with unrelated mean relative effects [13]. For our method, we tried to assess the overall inconsistency in the network according to the magnitude of potential inconsistency, for which a global testing approach was appropriate. Our sensitivity analysis was based on the idea of data imputation for missing contrasts in a star-shaped network, which requires limiting the number of loops to be closed. We therefore adopted an inconsistency model with unrelated mean relative effects, rather than a model estimating inconsistency factors, which is not recommended unless the number of closed loops is sufficiently large [13].

In the smoking cessation example, we showed that the sensitivity analysis may successfully simulate some expected results from an unknown complete network. In the full network, including all 24 RCTs, the estimated absolute extent of inconsistency for the contrasts ranged from 0.17 to 1.7 [16]. In our sensitivity analysis, the maximum obtained value assumed to be common for all contrasts was 1.05, a value in the middle of the above range. Regarding the robustness of the results of the star-shaped network, we could conclude that in 33% of the sensitivity analyses undertaken with statistically acceptable inconsistency, the resulting treatment ranking would be inconsistent with the ranking from the star-shaped network. These results suggest that a star-shaped network meta-analysis should be interpreted with caution unless the obtained treatment ranking is shown to be robust to uncertainty of the unverifiable consistency assumption.

In the application to simulated datasets, we demonstrated the sensitivity of the results after data imputation against the synthesis results from a given star-shaped network with different levels of within- and between-study variability. In a network meta-analysis, both inconsistency and heterogeneity can be caused by some common sources, such as differences in some effect modifiers, which are closely related to each other [13]. For this reason, performing a star-shaped network meta-analysis using a consistency model may be considered more valid when a lower level of heterogeneity within the network is present.

When we considered a star-shaped network with more than three interventions, we assumed that ${\overline{\omega}}_{jk}$ for *j* = *T*_2_, ⋯, *T*_*p* − 1_ and *k* = *T*_3_, ⋯, *T*_*p*_ (*j* ≠ *k*) would be simultaneously changed by an identical magnitude from 0 in their respective directions. However, it is also possible to assign different levels of inconsistency if there is an appropriate rationale for doing so. For example, in the full known complete network of the smoking cessation meta-analysis, there was a contrast for which the inconsistency estimate was observed to be somewhat larger than others, although no statistically significant inconsistency was found overall. If prior information was available on the diversity of the extent of inconsistency for the contrasts, taking such considerations into account may point to ways to further refine how to undertake a sensitivity analysis.

Some limitations of this study motivate further research. First, the estimated variance of each individual trial was treated as if it were the true variance in the network meta-analysis models in our approach. However, the variances themselves are given in the form of estimates, and it therefore might be necessary to consider uncertainty in the variances [43, 44]. A further investigation to introduce a probability distribution for the estimated variances would be worthwhile. Second, we used a point estimate of heterogeneity from a star-shaped network meta-analysis for the data imputation process. However, further research may consider generating the estimate of heterogeneity from its posterior distribution. Third, we built up a method that can be applied to a general form of comparative measure that follows at least asymptotic normality. This assumes using a log transformation for a ratio type of measure, such as log odds ratios or log relative risks, when a binary outcome was considered. However, since there is a correlation between log odds ratios (or log risk ratios) and their estimated variances, there could be an issue on pooling the estimates by the inverse variance weight method. An arm-specific data imputation strategy with arm-based modeling that accounts for specific types of outcome measures could also be considered for an elaboration of our method.

We defined consistency in the ranking as an unchanged order of the originally observed ranking. However, a change of ranking may not necessarily be interpreted as indicating an inconsistency in the results, depending on the probability difference based on which the order was obtained. Although the observed ranks were switched between treatments, their associated probabilities of being the best treatment might not be considered significantly different, as we observed from the overlapping distributions of probabilities in Additional file 3: Figure S5 for the smoking cessation example. However, it is a convention that authors report treatment rankings based only on the order of probabilities, and we tried to demonstrate how likely it was for the originally obtained conclusion from a star-shaped network to remain robust in terms of the order of rankings that authors would report.

An approach known as ‘threshold analysis’, based on a similar conceptual framework of sensitivity analysis to assess confidence in recommendations obtained from network meta-analyses, has been proposed in the literature [45–47]. Threshold analysis derives a set of thresholds that describe how much each data point from a study or contrast could change before the recommendation changes. This method could also be applied to a star-shaped network, such as the example created from the smoking cessation meta-analysis. Figure S6 in Additional file 3 presents results from the threshold analysis for the star-shaped network at the contrast level. If the invariant interval is within the 95% credible interval of the effect size for each contrast from a base-case star-shaped network meta-analysis in this context, it is interpreted that the optimal treatment recommendation could change. The result suggests some possibility of IC being optimal, instead of GC. Since only one study was available in the analysis in which GC was compared to NI, a wide credible interval for their relative effects was produced. As a result, the sensitivity analysis suggests that some potential change in the effect size estimate from its currently observed value—even within the range of the credible interval—could have changed the current recommendation to the second best option, IC.

In contrast with the results from the threshold analysis method, our approach suggested that the ranking of GC as distinctly more effective than other treatments would remain stable, whereas the rankings of IC and NI may be switched. Although both approaches utilize sensitivity analysis, they were designed to incorporate different concerns: the impact of potential bias in the given direct data or the impact of potential inconsistency between observed indirect evidence and non-existing direct data. The discrepancy in the results may stem from the fact that these approaches focus on different features.

Where individual patient data (IPD) are available for at least one of the trials included in a star-shaped network meta-analysis, methods for population-adjusted indirect comparisons, such as the matching-adjusted indirect comparison and the simulated indirect comparison, could be applied with improving balance in patient characteristics between the trials [48–50]. These population adjustment methods apply both to anchored comparisons and unanchored comparisons without a common comparator [51]. If there is a lack of overlap between IPD and aggregate data populations, it is necessary to assess the robustness of the comparisons because these methods may produce biased estimates, and our proposed method of sensitivity analysis will be a useful tool. Furthermore, when no IPD are accessible and if it is determined that the studies are highly exchangeable, researchers may just attempt to integrate data through a network meta-analysis using a consistency model. Our proposed method could serve as an alternative approach to assess the reliability of results from a star-shaped network before making a conclusion relying on those results.

## Conclusions

Our method will serve as a practical technique to investigate the reliability of results from star-shaped network meta-analyses under the unverifiable consistency assumption, and therefore will help to assess evidence for use in unbiased clinical decision-making.

## Supplementary Information

**Additional file 1: Appendix 1.** Descriptions of consistency and inconsistency models for a simple network with a closed loop consisting of interventions A, B, and C. **Appendix 2.** Details of how to derive the formula for calculating $\hat{\mathit{\operatorname{var}}}\left({\hat{\theta}}_{kBC}^{\ast}\right),k=1,\dots, l.$ 푙

**Additional file 2: Table S1.** Results of checking the inconsistency reported in the original analyses for smoking cessation data. Adapted from Lu and Ades, 2006 [11], and Dias et al., 2010 [16]. **Table S2.** Simulated datasets**. Table S3.** Results of a network meta-analysis using the complete network and derived star-shaped network for the smoking cessation data.

**Additional file 3: Figure S1.** Residual deviances by model type (y-axis) against the absolute extent of inconsistency (x-axis) according to the number of imputations in the smoking cessation example. **Figure S2.** Example for Crohn’s disease. **Figure S3.** Residual deviances by model type (y-axis) against the absolute extent of inconsistency (x-axis) for each simulated data set. **Figure S4.** Residual deviances by model type (y-axis) against the absolute extent of inconsistency (x-axis) according to the number of imputations in the simulated data set with *I*^2^ of 0% and a standard error of 1. **Figure S5.** Box-plots of the resulting probabilities of each intervention being the best from multiple imputation for the four interventions. **Figure S6**. Contrast-level forest plot with invariant intervals for the smoking cessation example.

## Data Availability

The datasets and R code generated during the current study are available online as an open-source project at https://github.com/yjh891114/Assessing-robustness-of-conclusion-from-a-star-shaped-network-meta-analysis-through-imputation.

## Abbreviations

RCT: Randomized controlled trial
NI: No intervention
SH: Self-help
IC: Individual counseling
GC: Group counseling
CrI: Credible interval
IPD: Individual patient data
