# Supplementary material for: A method for assessing robustness of the results of a star-shaped network meta-analysis under the unidentifiable consistency assumption
Source: BMC Med Res Methodol. 2021 Jun 1;21:113. doi: 10.1186/s12874-021-01290-1 (PMC8171049; doi:10.1186/s12874-021-01290-1)
Supplement: Supplementary file 1 — Additional file 1: Appendix 1. Descriptions of consistency and inconsistency models for a simple network with a closed loop consisting of interventions A, B, and C. Appendix 2. Details of how to derive the formula for calculating $\hat{\mathit{\operatorname{var}}}\left({\hat{\theta}}_{kBC}^{\ast}\right),k=1,\dots, l.$var^(θ^kBC∗),k=1,…,l. 푙 [file 12874_2021_1290_MOESM1_ESM.pdf]

Appendix 1. Descriptions of consistency and inconsistency models for a simple network with a closed loop consisting of interventions A, B, and C.

The data consist of $\hat{\theta}_{ijk}$, the observed relative effect size of treatment k compared to treatment j from study i $(j = A, B, k = B, C,\ j \neq k)$, and its variance, $\widehat{var}(\hat{\theta}_{ijk})$. The notation is identical to that used in the main body.

- Consistency model

  $\hat{\theta}_{ijk} \sim\ N\left(\theta_{ijk}, \widehat{var}(\hat{\theta}_{ijk})\right)$

  $\theta_{ijk} \sim Normal(d_{jk},\ \tau^2)$

  $\theta_{iAB} \sim Normal(d_{AB}, \tau^2),\ \theta_{iAC} \sim Normal(d_{AC}, \tau^2),\ \theta_{iBC} \sim Normal(d_{AC} - d_{AB}, \tau^2)$

  $d_{AB} \sim Normal(0{,}10000),\ d_{AC} \sim Normal(0{,}10000),\ \tau \sim Uniform(0, 5)$

- Inconsistency model

  $\hat{\theta}_{ijk} \sim\ N\left(\theta_{ijk}, \widehat{var}(\hat{\theta}_{ijk})\right)$

  $\theta_{ijk} \sim Normal(d_{jk},\ \tau^2)$

  $\theta_{iAB} \sim Normal(d_{AB}, \tau^2),\ \theta_{iAC} \sim Normal(d_{AC}, \tau^2),\ \theta_{iBC} \sim Normal(d_{BC}, \tau^2)$

  $d_{AB} \sim Normal(0{,}10000),\ d_{AC} \sim Normal(0{,}10000),\ d_{BC} \sim Normal(0{,}10000),$

  $\tau \sim Uniform(0, 5)$

Appendix 2. Details of how to derive the formula for calculating $\widehat{var}(\hat{\theta}^*_{kBC})$, k= $1, \ldots, l$.

The imputed data consisted of a number of treatment effect sizes of treatment C compared to B and their variances, $\hat{\theta}^*_{kBC}$ and $\widehat{var}(\hat{\theta}^*_{kBC})$, k= $1, \ldots, l$ (where l is the number of missing trials for comparison between B and C). The estimated variance of a pooled effect size from a pairwise random-effects meta-analysis of the data is $\frac{1}{\sum_{k=1}^{l} \frac{1}{\widehat{var}(\hat{\theta}^*_{kBC}) + \hat{\tau}^2_{BC}}}$, where $\hat{\tau}^2_{BC}$ is the extent of heterogeneity of the effect size [1].

Based on conditions II and III, we conservatively assumed that this variance would be the same as the variance of the effect size estimated from the original star-shaped network meta-analysis, $\widehat{var}(\hat{d}_{AC} - \hat{d}_{AB})$, and also assumed that $\hat{\tau}^2_{BC}$ would be equal to that of the overall heterogeneity across contrasts in the star-shaped network, $\hat{\tau}^2$. Accordingly, the following equation was established:

$$\widehat{var}\left(\hat{d}_{AC} - \hat{d}_{AB}\right) = \frac{1}{\sum_{k=1}^{l} \frac{1}{\widehat{var}(\hat{\theta}^*_{kBC}) + \hat{\tau}^2}} .$$

For simplicity, $\widehat{var}(\hat{\theta}^*_{1BC}), \cdots, \widehat{var}(\hat{\theta}^*_{lBC})$ were set up to be identical. The equation was thus expressed as:

$$\widehat{var}\left(\hat{d}_{AC} - \hat{d}_{AB}\right) = \frac{1}{\frac{l}{\widehat{var}(\hat{\theta}^*_{kBC}) + \hat{\tau}^2}}.$$

Finally, from the above equation, we obtained a formula, $\widehat{var}(\hat{\theta}^*_{kBC}) = l \cdot \widehat{var}(\hat{d}_{AC} - \hat{d}_{AB}) - \hat{\tau}^2$.

1. Whitehead A, Whitehead J: **A general parametric approach to the meta-analysis of randomized clinical trials**. *Statistics in medicine* 1991, **10**(11):1665-1677.
