# Supplementary material for: A method for assessing robustness of the results of a star-shaped network meta-analysis under the unidentifiable consistency assumption
Source: BMC Med Res Methodol. 2021 Jun 1;21:113. doi: 10.1186/s12874-021-01290-1 (PMC8171049; doi:10.1186/s12874-021-01290-1)
Supplement: Supplementary file 2 — Additional file 2: Table S1. Results of checking the inconsistency reported in the original analyses for smoking cessation data. Adapted from Lu and Ades, 2006 [11], and Dias et al., 2010 [16]. Table S2. Simulated datasets. Table S3. Results of a network meta-analysis using the complete network and derived star-shaped network for the smoking cessation data. [file 12874_2021_1290_MOESM2_ESM.pdf]

Table S1. Results of checking the inconsistency reported in the original analyses for smoking cessation data. Adapted from Lu and Ades, 2006 [11], and Dias et al., 2010 [16].

	Effect size estimate (sd)			
Comparisons	MTC [11]	Direct [16]	Indirect [16]	P- value [16]
NI versus SH	0.449 (0.441)	0.342 (0.55)	0.706 (0.635)	0.65
NI versus IC	0.860 (0.248)	0.845 (0.254)	0.673 (0.679)	0.79
NI versus GC	1.088 (0.516)	1.360 (0.829)	1.108 (0.539)	0.81
SH versus IC	0.258 (0.519	-0.052 (0.702)	0.519 (0.503)	0.49
SH versus GC	0.636 (0.546	0.676 (0.698)	0.511 (0.684)	0.85
IC versus GC	0.226 (0.545)	-0.085(0.479)	1.708 (0.893)	0.07
Variance of inconsistency factor (σ_ω^2)	0.61			
Variance of heterogeneity (τ^2)	0.78			
P($\sigma_\omega^2 > \tau^2$)	0.226			

NI, no intervention; SH, self-help; IC, individual counseling; GC, group counseling; P- value, Bayesian p-value measuring agreement between direct and indirect estimates for each split node; MTC, mixed treatment comparison;

Table S2. Simulated datasets

			I^2		
			0%	40%	70%
Standard error	Study	Comparison	Effect size	Effect size	Effect size
1	1	A vs B	0.5	-1	-1.7
	2	A vs B	0.5	-0.5	-1
	3	A vs B	0.5	0.5	0.5
	4	A vs B	0.5	1.5	2
	5	A vs B	0.5	2	2.7
	6	A vs C	1	-0.5	-1.2
	7	A vs C	1	0	-0.5
	8	A vs C	1	1	1
	9	A vs C	1	2	2.5
	10	A vs C	1	2.5	3.2
Standard error	Study	Comparison	Effect size	Effect size	Effect size
2	1	A vs B	0.5	-2.5	-4
	2	A vs B	0.5	-1.5	-2
	3	A vs B	0.5	0.5	0.5
	4	A vs B	0.5	2.5	3
	5	A vs B	0.5	3.5	5
	6	A vs C	1	-2	-3.5
	7	A vs C	1	-1	-1.5
	8	A vs C	1	1	1
	9	A vs C	1	3	3.5
	10	A vs C	1	4	5.5
Standard error	Study	Comparison	Effect size	Effect size	Effect size
$2\sqrt{2}$	1	A vs B	0.5	-4	-5.5
	2	A vs B	0.5	-2	-3.5
	3	A vs B	0.5	0.5	0.5
	4	A vs B	0.5	3	4.5
	5	A vs B	0.5	5	6.5
	6	A vs C	1	-3.5	-5
	7	A vs C	1	-1.5	-3
	8	A vs C	1	1	1
	9	A vs C	1	3.5	5
	10	A vs C	1	5.5	7

Table S3. Results of a network meta-analysis using the complete network and derived star-shaped network for the smoking cessation data

	Parameter	Complete network		Star-shaped network	
		Estimate (95% CrI)	P(best)	Estimate (95% CrI)	P(best)
Treatment					
A: NI	-		0.00		0.00
B: SH	d_{AB}	0.43 (-0.38, 1.25)	0.04	0.33 (-0.73, 1.39)	0.02
C: IC	d_{AC}	0.73 (0.26, 1.20)	0.10	0.72 (0.19, 1.25)	0.04
D: GC	d_{AD}	1.38 (0.25, 2.50)	0.86	3.52 (0.12, 6.93)	0.94
Heterogeneity	τ	0.78		0.85	

CrI, credible interval; NI, no intervention; SH, self-help; IC, individual counseling; GC, group counseling.
