# Supplementary material for: A method for assessing robustness of the results of a star-shaped network meta-analysis under the unidentifiable consistency assumption
Source: BMC Med Res Methodol. 2021 Jun 1;21:113. doi: 10.1186/s12874-021-01290-1 (PMC8171049; doi:10.1186/s12874-021-01290-1)
Supplement: Supplementary file 3 — Additional file 3: Figure S1. Residual deviances by model type (y-axis) against the absolute extent of inconsistency (x-axis) according to the number of imputations in the smoking cessation example. Figure S2. Example for Crohn’s disease. Figure S3. Residual deviances by model type (y-axis) against the absolute extent of inconsistency (x-axis) for each simulated data set. Figure S4. Residual deviances by model type (y-axis) against the absolute extent of inconsistency (x-axis) according to the number of imputations in the simulated data set with I2 of 0% and a standard error of 1. Figure S5. Box-plots of the resulting probabilities of each intervention being the best from multiple imputation for the four interventions. Figure S6. Contrast-level forest plot with invariant intervals for the smoking cessation example. [file 12874_2021_1290_MOESM3_ESM.pdf]

Figure S1. Residual deviances by model type (y-axis) against the absolute extent of inconsistency (x-axis) according to the number of imputations in the smoking cessation example.

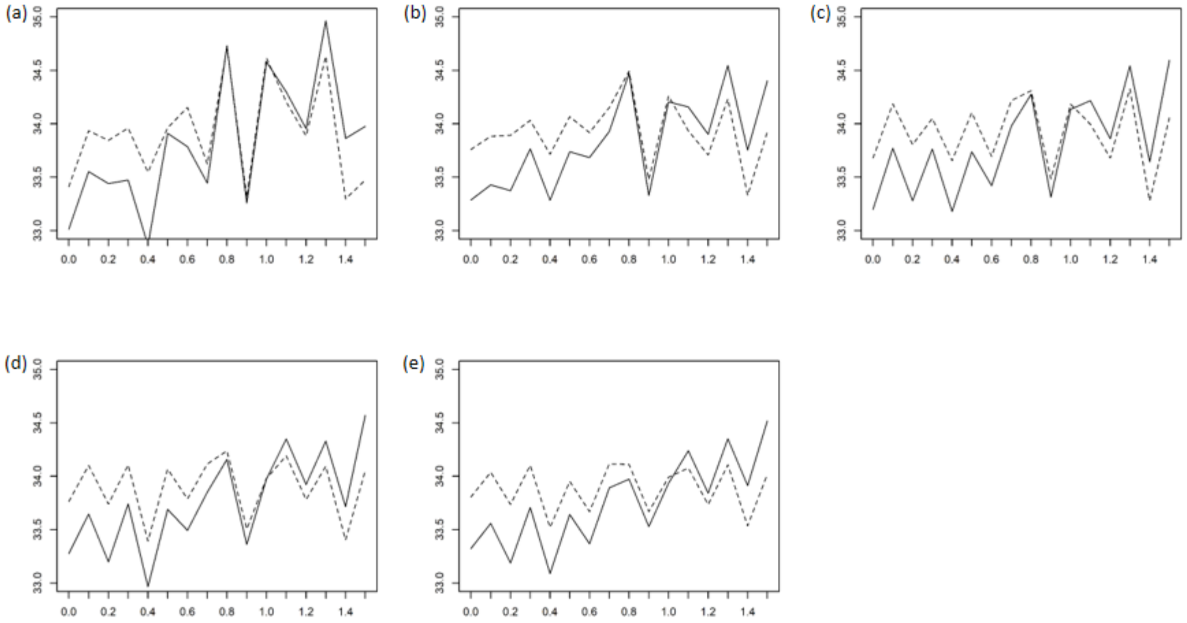

(a) When the number of imputations is 100, (b) when the number of imputations is 200, (c) when the number of imputations is 300, (d) when the number of imputations is 400, and (e) when the number of imputations is 500. The solid line and dashed line indicate the consistency model and inconsistency model, respectively.

Figure S2. Example for Crohn's disease.

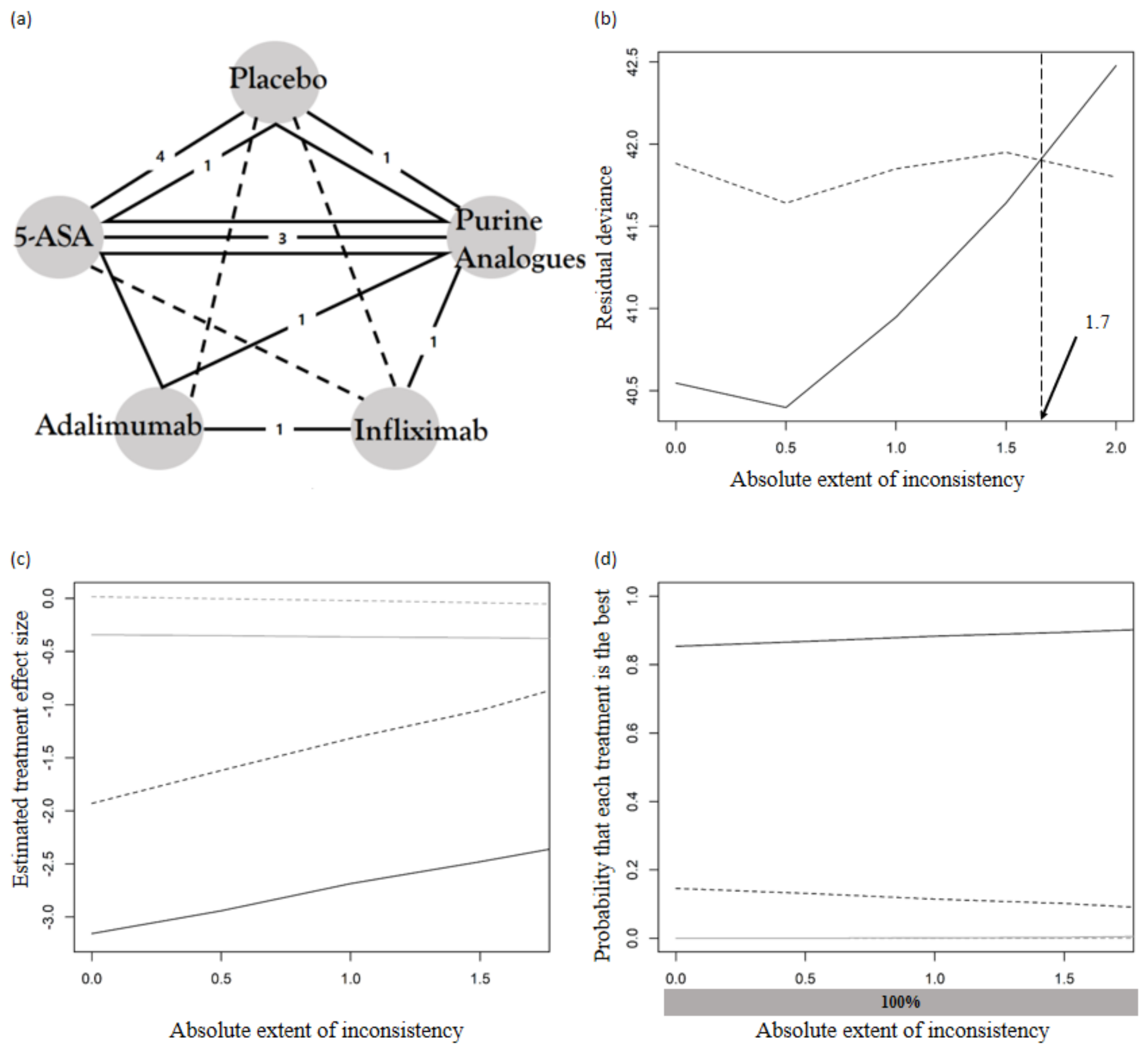

(a) An evidence network for Crohn's disease. Solid lines indicate existing head-to-head comparisons in randomized controlled trials (RCT) and triangles represent three arm RCTs. Dashed lines indicate imputed direct comparisons. The number of trials are presented within the lines. 5-ASA, 5-aminosalicylic acid. (b) Residual deviance by model type against the absolute extent of inconsistency. The solid line and dashed line indicate the consistency model and inconsistency model, respectively. A vertical line marks the point at which the two lines cross, and the value of that point on the x-axis is shown. (c) Trend plot of estimates of basic

parameters against the extent of inconsistency within the obtained range. Black, dotted black, dim gray, and dotted dim gray lines indicate the estimated treatment effect sizes for adalimumab, infliximab, 5-ASA, and placebo compared to purine analogues, respectively. (d) The probability that each treatment is the best for reducing relapse risk against the extent of inconsistency within the obtained range. The back, dotted black, dim gray, dotted dim gray, and dashed dim gray lines indicate the estimated probabilities corresponding to adalimumab, infliximab, 5-ASA, placebo, and purine analogues, respectively, and the percentage in the dark gray box represents the proportion of inconsistency that resulted in a consistent ranking of treatments relative to the original ranking, respectively.

Figure S3. Residual deviances by model type (y-axis) against the absolute extent of inconsistency (x-axis) for each simulated data set.

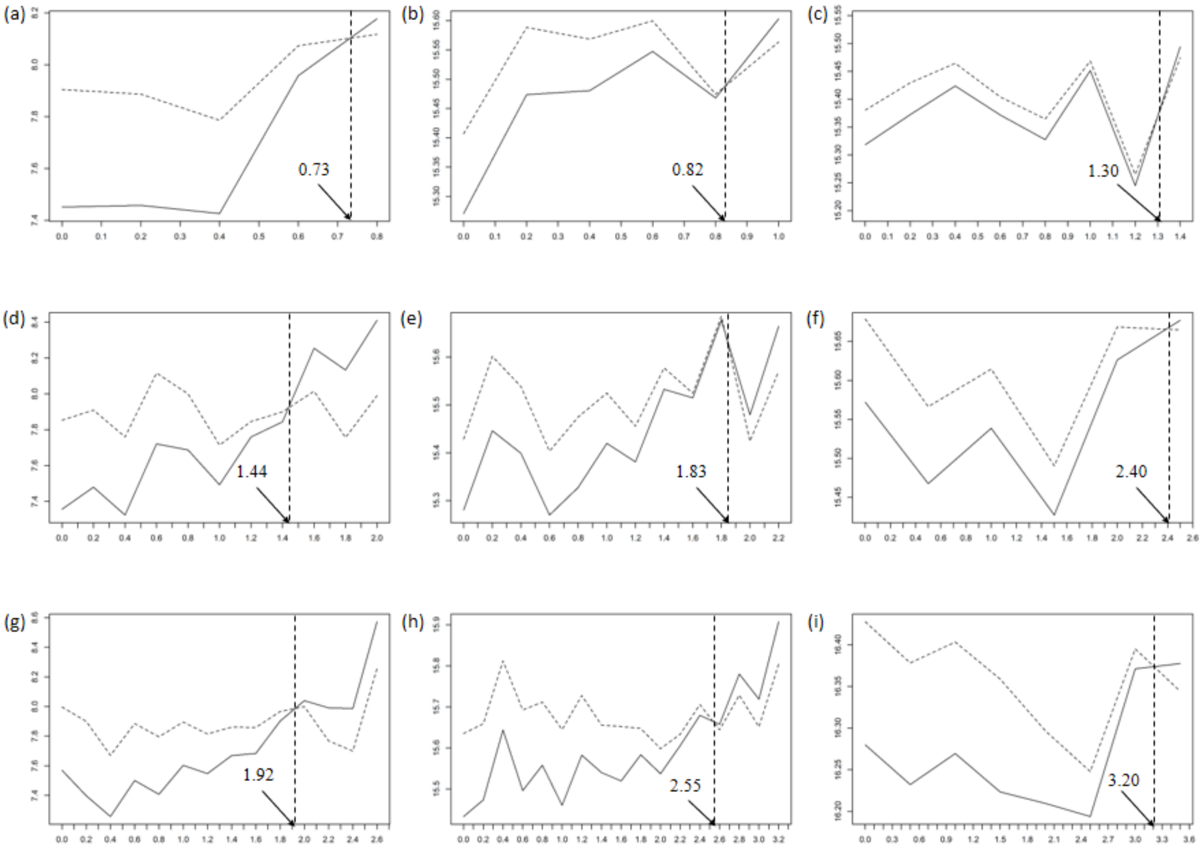

(a) When I^2 is 0% and the standard error is 1, (b) when I^2 is 40% and the standard error is 1, (c) when I^2 is 70% and the standard error is 1, (d) when I^2 is 0% and the standard error is 2, (e) when I^2 is 40% and the standard error is 2, (f) when I^2 is 70% and the standard error is 2, (g) when I^2 is 0% and the standard error is $2\sqrt{2}$, (h) when I^2 is 40% and the standard error is $2\sqrt{2}$, and (i) when I^2 is 70% and the standard error is $2\sqrt{2}$. The solid line and dashed line indicate the consistency model and inconsistency model, respectively. A vertical line marks the point at which the two lines cross.

Figure S4. Residual deviances by model type (y-axis) against the absolute extent of inconsistency (x-axis) according to the number of imputations in the simulated data set with I^2 of 0% and a standard error of 1.

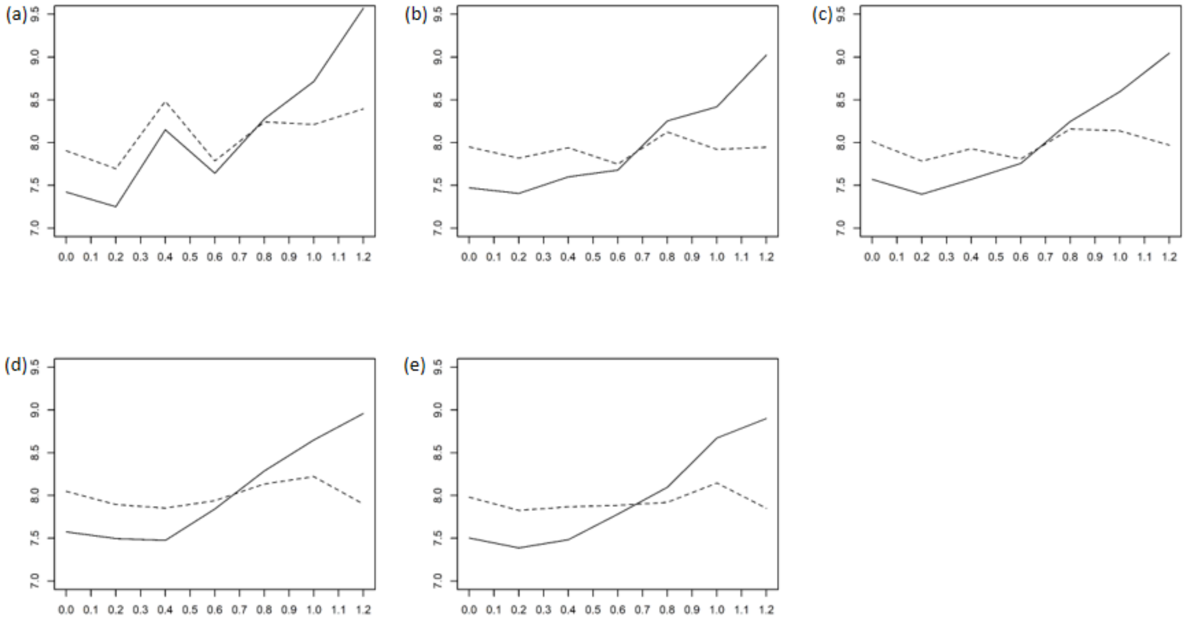

(a) When the number of imputations is 100, (b) when the number of imputations is 200, (c) when the number of imputations is 300, (d) when the number of imputations is 400, and (e) when the number of imputations is 500. The solid line and dashed line indicate the consistency model and inconsistency model, respectively.

Figure S5. Box-plots of the resulting probabilities of each intervention being the best from multiple imputation for the four interventions

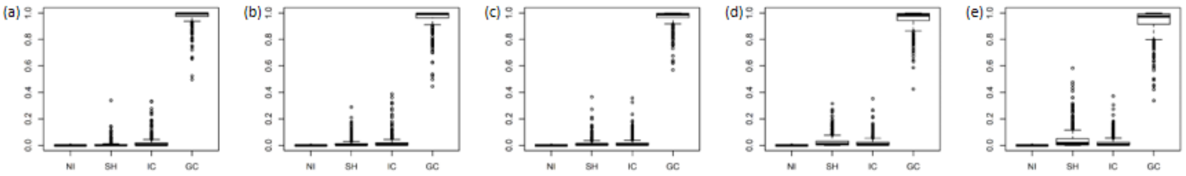

(a) When the absolute extent of inconsistency is 0.2, (b) when the absolute extent of inconsistency is 0.4, (c) when the absolute extent of inconsistency is 0.6, (d) when the absolute extent of inconsistency is 0.8, and (e) when the absolute extent of inconsistency is 1.0. NI, no intervention; SH, self-help; IC, individual counseling; GC, group counseling.

Figure S6. Contrast-level forest plot with invariant intervals for the smoking cessation example

Contrast	Mean	95% Credible Interval	Invariant Interval		
SH vs NI	0.33	(-0.74, 1.41)	–	(NT, 3.49)	SH
IC vs NI	0.72	(0.21, 1.28)	SH	(-45.93, 3.73)	IC
**GC vs NI**	3.52	(0.14, 6.97)	IC	(0.72, NT)	–

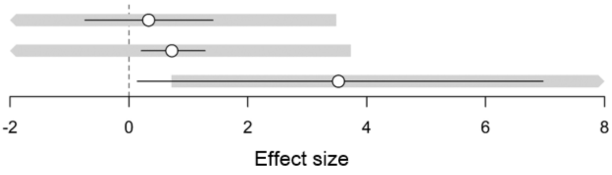

The base-case optimal treatment is GC. Bold letters in the table emphasize contrast estimates with short invariant intervals lying within the 95% credible interval. NI, no intervention; SH, self-help; IC, individual counseling; GC, group counseling.
